# Voxel-based morphometry of disgust sensitivity

**DOI:** 10.1080/17470919.2017.1288657

**Published:** 2017-02-06

**Authors:** Albert Wabnegger, Sonja Übel, Anne Schienle

**Affiliations:** ^a^ Department of Psychology, Section Clinical Psychology, University of Graz, BioTechMedGraz, Graz, Austria

**Keywords:** Emotion regulation, disgust sensitivity, voxel-based morphometry

## Abstract

**Background**: Difficulties with the regulation of negative affect have been extensively studied in neuroimaging research. However, dysregulation of a specific emotion, disgust, has hardly been investigated. In the present study, we used voxel-based morphometry to identify whether gray matter volume (GMV) of frontal regions is correlated with personality traits associated with general and disgust-specific emotion regulation difficulties.

**Method**: We analyzed T1-weighted images of 49 females (mean age = 22.8 years, SD = 3.2). The women rated their disgust sensitivity (DS) (temporally stable tendency to experience disgust as uncontrollable and extremely aversive) as well as general difficulties with emotion regulation.

**Results**: DS and general emotion regulation deficits were positively associated with a heightened GMV of the orbitofrontal cortex. DS additionally showed a negative association with GMV of the medial prefrontal cortex.

**Conclusion**: The present study revealed shared and distinct contributions of frontal brain regions to disgust-specific and general emotion regulation difficulties.

## Introduction

The basic emotion of disgust serves as a disease-avoidance mechanism and is triggered by stimuli which are able to contaminate objects and people (Oaten, Stevenson, & Case, 2009). For example, body secretions and spoiled food are considered core disgust elicitors, which evoke this emotion in everyone. However, there are individual differences in the intensity of experienced disgust. These differences are related to the personality trait disgust proneness, which is defined as the temporally stable tendency of a person to experience disgust across a variety of situations (e.g., when exposed to the smell of spoiled milk). Individuals high in disgust proneness tend to react more frequently and more intensely with disgust to different aversive stimuli (Schienle, Walter, Stark, & Vaitl, 2002). Disgust proneness has widely been studied in functional and structural neuroimaging research in clinical and healthy samples (e.g., Ille et al., 2015; Schäfer, Leutgeb, Reishofer, Ebner, & Schienle, 2009; Scharmüller & Schienle, 2012; Schienle, Übel, Schöngaßner, Ille, & Scharmüller, 2013; Watkins et al., 2015). A functional magnetic resonance imaging study from Schäfer et al. (2009) found a positive correlation between activation in the orbitofrontal cortex (OFC) and the insula and self-reported disgust proneness in healthy women who looked at disgust-eliciting scenes (e.g., maggots, dirty toilet). Furthermore, disgust proneness has also been studied in clinical samples. For instance, Ille et al. (2015) found a positive correlation between OFC volume and the olfactory-associated disgust proneness in patients with Parkinson’s disease.

However, neuroimaging studies on a second disgust-related personality trait disgust sensitivity (DS) are still rare. DS refers to the negative evaluation of one’s own disgust symptoms (e.g., nausea) and problems in disgust regulation (Olatunji, Cisler, Deacon, Connoly, & Lohr, 2007; Schienle, Dietmaier, Ille, & Leutgeb, 2010). Individuals who score high on DS questionnaires (e.g., Schienle et al., 2010) have problems to control their disgust feelings and experience shame and embarrassment because of their own disgust symptoms. As indicated by a previous study, DS can be understood as a specific type of emotion regulation deficit (Cisler, Olatunji, & Lohr, 2008). In this study, participants with high DS scores reported difficulties with the control and the acceptance of aversive affective states, including disgust. Furthermore, DS is relevant for different psychopathologies (e.g., phobias). DS is positively associated with the fear of injections and with social anxiety. For instance, it has been argued that deficits in disgust regulation may lead to fainting while getting an injection (Cisler et al., 2008; Olatunji et al., 2007). Finally, in the validation sample for the two disgust questionnaires (Schienle et al., 2010), DS and disgust proneness were only moderately correlated (*r* = 0.34). Hence, both constructs can clearly be distinguished from each other.

On a neurofunctional basis, Schäfer et al. (2009) observed negative correlations between activity in brain areas crucial for emotion regulation, such as the medial prefrontal cortex and dorsolateral prefrontal cortex (mPFC, DLPFC) and DS. To the best of our knowledge, the association between brain structure and DS had not been analyzed before.

In contrast to disgust regulation, downregulation of general negative affect has widely been studied (e.g., Cutuli, 2014; Giuliani, Drabant, Bhatnagar, & Gross, 2011; Kong et al., 2014; Mak, Wong, Han, & Lee, 2009). Brain imaging studies found especially frontal regions (e.g., OFC, mPFC) and the anterior cingulate cortex (ACC) to be involved in emotion regulation (e.g., Beauregard, Paquette, & Levesque, 2006; Etkin, Prater, Hoeft, Menon, & Schatzberg, 2010; Phan et al., 2005; Urry et al., 2006). For instance, Welborn et al. (2009) reported that the volume of the mPFC was positively associated with the emotion regulation strategy of reappraisal in a healthy sample. The downregulation ability for sadness was positively associated with activity of the OFC (Beauregard et al., 2006). This result is in line with the observation that the OFC constitutes one key region for the regulation of emotions (Kringelbach & Rolls, 2004).

Therefore, in the present voxel-based morphometry study (VBM), we sought to identify associations between gray matter volume (GMV) of regions implicated in emotion regulation (e.g., OFC, ACC) and the personality trait DS. General difficulties in emotion regulation were assessed as a control variable.

## Methods

### Participants

We investigated data from 49 healthy females with a mean age of *M* = 22.76 years (SD = 3.19). All participants were free from psychotropic medication, somatic problems and mental disorders as assured by the Brief Symptom Inventory (Derogatis, 1993). The BSI is a 53-item questionnaire covering nine symptom dimensions (e.g., depression, anxiety). Respondents rank each item on a 5-point scale ranging from 0 (not at all) to 4 (extremely). The global severity index (GSI) can be calculated as index of overall psychological distress with a critical cutoff value of GSI ≥ 63 points (*T*-value). In our sample, the scores ranged between 27 and 48. Written informed consent was obtained from all subjects. Participants were recruited via local newspaper advertisements and via flyers handed out on the Campus of the University of Graz. The study was conducted in accordance with the Declaration of Helsinki and was reviewed by the ethics committee of the Medical University of Graz.

### Questionnaires

The participants completed the Scale for the Assessment of Disgust Sensitivity (SADS; Schienle et al., 2010) and the questionnaire for Difficulties in Emotion Regulation (DERS; Gratz & Roemer, 2004). The SADS measures controlling and appraisal of one’s own disgust feelings (e.g., “I feel embarrassed, when someone recognizes my discomposure in disgusting situations”; “I try not to encounter disgusting situations, because I am afraid of not being able to control the feeling”) on seven items rated on a 5-point Likert scale (0 = “never true”, 5 = “always true”). Higher scores indicate a higher DS. The Cronbach’s alpha of the SADS is 0.85.

The DERS measures emotional dysregulation (e.g., “When I’m upset, I feel ashamed with myself for feeling that way”) and consists of 36 items that have to be rated on a 5-point Likert scale (1 = “almost never”, 5 = “almost always”). Higher scores indicate greater regulation difficulties. The Cronbach’s alpha of the scale is 0.93.

### Image acquisition and VBM analysis

T1-weighted scans were acquired using a 3-T Siemens Skyra with a 32-channel head coil (Siemens, Erlangen, Germany). The scanning parameters were as follows: voxel size: 0.88 × 0.88 × 0.88 mm; 192 transverse slices, FoV = 224 mm, slice thickness: 0.88 mm, TE = 1.89 ms, TR = 1680 ms; TI = 1000 ms, flip-angle = 8°.

Structural scans were analyzed with the Computational Anatomy Toolbox (CAT12; r864) implemented in SPM12 (v6685; Wellcome Trust Centre for Neuroimaging; http://www.fil.ion.ucl.ac.uk/spm/software/spm12/) in order to gain voxel-wise comparisons of GMV.

Prior to the normalization procedure, each individual was co-registered to the “avg305T1-template” in SPM12 using normalized mutual information. This approach should replace the procedure of manually repositioning of each scan. First structural data were segmented into gray and white matter as well as cerebrospinal fluid. We applied mainly default settings of the CAT12 toolbox. To compensate for the effect of spatial normalization, images were modulated, as spatial normalization could lead to volume changes. This approach preserves the total amount of gray matter. The final resulting voxel size was 1.5 × 1.5 × 1.5 mm. For quality assurance, we checked resulting images for homogeneity. As all images had high correlation values (>0.88); none of the images had to be discarded. Finally, gray matter images were smoothed with a Gaussian kernel with a full width at half maximum (FWHM) of 8 mm.

Statistical analyses were carried out using random effects models. Questionnaire scores were correlated via multiple regressions with GMV. Additionally, the total intracranial volume was added as a covariate of no interest, in order to correct for different brain sizes. To restrict analysis to gray matter, images were thresholded for all analyses with an absolute threshold of 0.1.

Based on previous studies on general and specific emotion regulation (e.g., Etkin et al., 2010; Ille et al., 2015; Longe et al., 2010; Schäfer et al., 2009), we selected the following regions of interest: OFC, mPFC, ACC. The current study used masks with a 50% threshold derived from the Harvard–Oxford cortical structural atlas Center for Morphometric Analysis, MGH-East, Boston/MA, USA). Additionally, masks were resliced to a voxel size of 1.5 × 1.5 × 1.5 mm with the nearest neighbor function. The detailed MNI-coordinate axis of the used masks is as follows: OFC L (*x* = −50 to −12; *y* = 7 to 35; *z* = −1 to −27); OFC R (*x* = 10–51; *y* = 7 – 32; *z* = −24 to −3); MPFC (*x* = −8 to 9; *y* = 31–54; *z* = −29 to −7); and ACC (*x* = −8 to 11; *y* = −15 to −44; *z* = −9 to 47).

We applied a cluster-building threshold of 0.005 uncorrected with an extent threshold of at least 20 contiguous voxels. Only results are reported when *p* value corrected for family-wise-error falls below 0.05 on peak level (small volume correction).

## Results

### Self-report data

Participants obtained a mean SADS score of *M* = 1.04 (SD = 0.80) and a mean DERS score of *M* = 2.26 (SD = 0.56). SADS and DERS were positively correlated (*r* = 0.35, *p* = 0.013).

### Voxel-based morphometry

**Figure 1.**
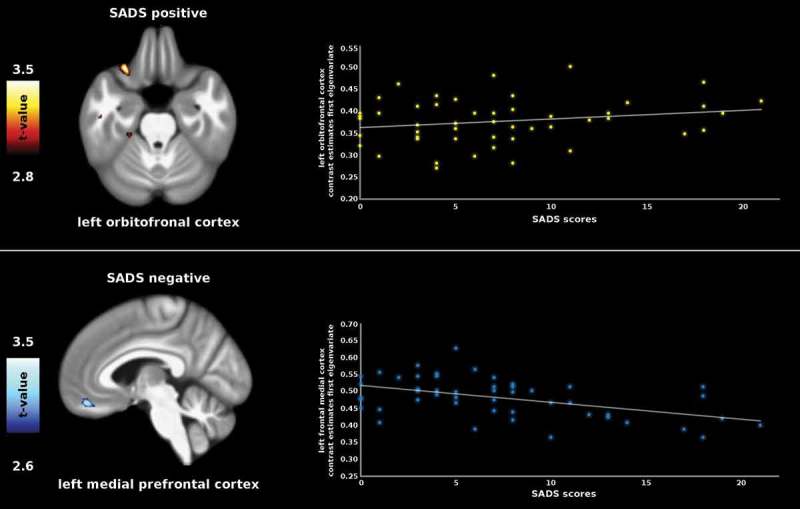
Correlation between SADS scores and gray matter volume of prefrontal regions.

SADS scores correlated positively with GMV of the left OFC and negatively with GMV of the (mPFC; see Figure 1). DERS scores correlated positively with GMV of the right OFC. For detailed information, please see Table 1.

**Table 1.** Results of the correlations between gray matter volume of regions of interest and self-report data.

Questionnaire		ROI	H	*x*	*y*	*z*	*t*	*p* (FWE)	CS
SADS									
	+	OFC	L	−29	26	−23	3.52	0.04	78
	−	mPFC	L	−6	44	−17	3.35	0.042	83
DERS									
	+	OFC	R	38	20	−17	3.41	0.047	237

**±: Direction of correlation; OFC: orbitofrontal cortex; mPFC: medial prefrontal cortex; H: hemisphere; *x*, *y*, *z*: MNI-coordinates; *p* (FWE): *p* value corrected for family-wise-error; CS: cluster size – number of voxels in associated cluster.**

## Discussion

In the present VBM study, we revealed common and distinct correlational patterns between GMV of frontal brain regions and DS as well as general emotion regulation capacity. DS correlated positively with GMV of the left OFC and negatively with the left medial frontal cortex. This means that greater difficulties with the regulation of disgust went along with enhanced GMV of the OFC and reduced GMV of the left mPFC.

In order to interpret these findings, it is important to note that the OFC does not constitute a uniform structure but can be divided into four sections: medial orbital, anterior orbital, posterior orbital and lateral orbital (Rolls, Joliot, & Tzourio-Mazoyer, 2015). An fMRI by Sturm et al. (2013) found that the lateral OFC was important for self-conscious emotions like shame. The participants underwent an embarrassing karaoke task, which elicited enhanced lateral OFC activation. The observed peak in the present study was also located in the lateral portion of the OFC. Several SADS items (indexing DS) deal with the experience of shame in disgust-relevant situations (e.g., “I feel embarrassed, when someone recognizes my discomfort in disgusting situations”). Especially, the inability to downregulate one’s own disgust feelings in social contexts can lead to shame (Schienle et al., 2010). For example, for many people, it is considered inacceptable to feel disgusted by another person (e.g., in the context of nursing care). Hence, our results suggest that a greater volume of the lateral OFC might increase one’s own susceptibility to react with shame in disgust-related situations.

However, also a slightly different interpretation is possible. The continuous evaluation of oneself in disgust-related situations could have led to an increase of GMV in the lateral OFC. Additionally, greater difficulties in general emotion regulation, as reflected by the overall DERS score, were accompanied by greater GMV of the right lateral OFC. In line, an fMRI study by Golkar et al. (2012) was able to show that lateral orbitofrontal activity could only be observed when the participants conducted emotion regulation during the presentation of negative pictures. A good portion of the items from the DERS deal with negative emotionality (e.g., “When I’m upset, I feel like I am weak”). Hence, we speculate that an enhanced volume of the lateral OFC might be associated with negative emotionality.

Furthermore, GMV of the left mPFC was negatively associated with DS, indexed by SADS scores. As pointed out by Wager et al. (2008), the mPFC is involved in the successful downregulation of negative emotions. In line, the present results indicate that greater GMV in the mPFC also facilitates the controlling of one’s own disgust feelings.

It is worth to mention that we found the same positive correlational pattern as Cisler et al. (2008) between DS and emotion regulation capacity indicating that both constructs not only share common variance but also own distinct characteristics.

We have to mention the following limitation of our study. We only investigated females, because of gender differences in emotion regulation strategies. Women more frequently report to use cognitive appraisal (i.e., reinterpretation of an emotion-eliciting situation) in contrast to men (Gross & John, 2003). Therefore, the present results cannot be generalized to men.

In conclusion, this is the first study that revealed a differential association between GMV of frontal regions and disgust regulation and nonspecific emotion regulation strategies on the trait level.
